# Exploration and Implementation of a Pre-Impact Fall Recognition Method Based on an Inertial Body Sensor Network

**DOI:** 10.3390/s121115338

**Published:** 2012-11-08

**Authors:** Guoru Zhao, Zhanyong Mei, Ding Liang, Kamen Ivanov, Yanwei Guo, Yongfeng Wang, Lei Wang

**Affiliations:** Shenzhen Key Laboratory for Low-cost Healthcare, and Shenzhen Institutes of Advanced Technology, Chinese Academy of Sciences, 1068 Xueyuan Road, Shenzhen 518055, China; E-Mails: gr.zhao@siat.ac.cn (G.Z.); zy.mei@siat.ac.cn (Z.M.); ding.liang@siat.ac.cn (D.L.); kamen@siat.ac.cn (K.I.); yw.guo@siat.ac.cn (Y.G.); wangyf@siat.ac.cn (Y.W.)

**Keywords:** biomechanics of fall, early pre-impact fall alarm, pre-impact lead time, postural instability, body sensor network

## Abstract

The unintentional injuries due to falls in elderly people give rise to a multitude of health and economic problems due to the growing aging population. The use of early pre-impact fall alarm and self-protective control could greatly reduce fall injuries. This paper aimed to explore and implement a pre-impact fall recognition/alarm method for free-direction fall activities based on understanding of the pre-impact lead time of falls and the angle of body postural stability using an inertial body sensor network. Eight healthy Asian adult subjects were arranged to perform three kinds of daily living activities and three kinds of fall activities. Nine MTx sensor modules were used to measure the body segmental kinematic characteristics of each subject for pre-impact fall recognition/alarm. Our analysis of the kinematic features of human body segments showed that the chest was the optimal sensor placement for an early pre-impact recognition/alarm (*i.e*., prediction/alarm of a fall event before it happens) and post-fall detection (*i.e.*, detection of a fall event after it already happened). Furthermore, by comparative analysis of threshold levels for acceleration and angular rate, two acceleration thresholds were determined for early pre-impact alarm (7 m/s/s) and post-fall detection (20 m/s/s) under experimental conditions. The critical angles of postural stability of torso segment in three kinds of fall activities (forward, sideway and backward fall) were determined as 23.9 ± 3.3, 49.9 ± 4.1 and 9.9 ± 2.5 degrees, respectively, and the relative average pre-impact lead times were 329 ± 21, 265 ± 35 and 257 ± 36 ms. The results implied that among the three fall activities the sideway fall was associated with the largest postural stability angle and the forward fall was associated with the longest time to adjust body angle to avoid the fall; the backward fall was the most difficult to avoid among the three kinds of fall events due to the toughest combination of shortest lead time and smallest angle of postural stability which made it difficult for the self-protective control mechanism to adjust the body in time to avoid falling down.

## 1. Introduction

Falls are the second leading cause of accidental injury deaths worldwide, and annually an estimated 424,000 individuals die globally from falls, of which over 80% occur in low- and middle-income countries [1]. Most patients with chronic illnesses (e.g., Parkinson's disease, stroke, arthritis and osteoporosis) and the elderly are at a higher risk of falling. The unintentional injuries due to falls give rise to a multitude of health and economic problems [2,3]. One of the most critical challenges faced by healthcare for the elderly is how to achieve early fall recognition/alarm to prevent falls and maintain safe standing and walking [4,5].

In recent years, many researchers have developed a series of methods for early pre-impact fall recognition/alarm. The methods included three main techniques: video-based sensing, ambient sensing and wearable sensing. The video-based technique is widely applied in healthcare; however its drawbacks are the high cost as well as some fears regarding the privacy of patients [6]. The equipment used for application of ambient sensing is typically installed on the floor or bed which militates against the convenience of this method [7]. By using the two methods above it is still very difficult to achieve early pre-impact fall recognition/alarm. Thus, the wearable sensing technique appears to be the most convenient for use in biomechanics of fall due to its low-cost, ease-of-use and technical advantages [8,9].

In terms of early fall recognition/alarm, one of the most important parameters is considered to be the pre-impact lead time, which refers to the ability of the measurement system to predict falls: optimal sensor placements and optimal method of measurement contribute to better fall recognition/alarm capability, and thus to a larger pre-impact lead time. Different kinds of fall activities are associated with different conditions to detect fall events, and thus, with different lead times.

One typical problem that prevents successful early pre-impact recognition/alarm appears to be the too-short pre-impact lead time. The longest known lead time was reported by Nyan and its duration determined using a video-based system was about 700 ms [10,11]; in [12] a pre-impact fall lead time of about 220 ms was obtained using a gyroscope and high speed camera. However, the methods above cannot be used for practical pre-impact fall recognition/alarm. In our past preliminary research [13], the best pre-impact lead time of fall achieved in lab experimental conditions was about 500 ms by using an inertial sensing method.

In order to collect enough information about free-direction falls, and thus further understand the fall postural instability and fall prevention mechanisms, the kinematic features of falls in different directions were explored in this paper. Firstly, the segmental kinematic characteristics of the human body were analyzed in order to find the optimal placements for sensors in three different fall activities based on a wearable inertial sensor network. Then, threshold levels were determined for fall recognition/alarm. The average pre-impact lead time and critical angle of body postural stability were calculated and analyzed in three different fall activities to understand the fall prevention mechanism. In summary, the main contributions of this paper are as follows:

- We presented a pre-impact fall recognition/alarm method that uses comparative and optimal approaches; it is based on an inertial body sensor network which consists of multiple nodes located on different segments of the human body. This method makes it possible to receive alarm early enough to reduce the risk of falls or completely prevent them.
- We explored the optimal sensor placement and the optimal threshold levels for pre-impact fall recognition/alarm by analyzing accelerometer and gyroscope data, as well as the orientation data from nine sensors located on different segments of the human body. The results showed that the chest was the optimal sensor placement for early pre-impact fall recognition/alarm, and it was better to use the acceleration as input parameter for early fall recognition/alarm rather than the angular rate.
- In our experiments we achieved the longest average lead time of 329 ± 21 ms during forward falls and the largest average angle of postural stability of 49.9 ± 4.1 degrees during sideway falls, which we consider a very good result. The results implied that, due to the specific trade-off between the pre-impact lead time and the angle of postural stability for each kind of events, the forward and sideway falls could be easily prevented, while it is difficult to avoid backward falls.

The rest of the paper is organized as follows: Section 2 gives an overview of the methods for fall detection that use inertial sensing technology. Section 3 presents our experimental design and methods. The experimental results and discussion are presented in Section 4. Section 5 concludes the paper and gives directions for future work.

## 2. State of the Art

The rapid development of wireless sensors, the low-cost, ease-of-use and wide availability make body sensor networks (BSN) an increasingly attractive solution for healthcare applications, such as health monitoring, gait analysis, activity recognition and fall detection.

Applications using body sensor networks are associated with different technical problems. Such are the matters related to accuracy, portability, lost data packet recovery, clock synchronization, sensor placement optimization, processing algorithms, data fusion, *etc*. Many researchers have searched for better solutions of these problems. Keally [14] presented a PBN solution for activity recognition feedback for mobile devices which was portable, lightweight, and accurate. Wark [15,16] presented a mobile sensor network for human motion monitoring and indoor localization. Liu [17] developed an efficient and accurate clock synchronization scheme for wireless sensor networks. Keally [18] explored how to use sensor collaboration to take advantage of sensor diversity in wireless sensor networks. Wu [19] explored a collision recovery method to decrease the packet losses in wireless sensor networks. Li [20] presented a fall detection algorithm using posture and context information that can reduce false positives.

In applications for fall detection based on inertial sensing technology, a single sensor on a single human body segment is usually used and detection is based on some pre-set threshold level. This approach is associated with ease-of-implementation and fast algorithm response. Such applications, based on a single accelerometer are described in [21–24]. Another example, based on a single gyroscope is described in [25]. However, these single-sensor methods were not accurate enough for fall detection. In order to improve the fall detection accuracy, a couple (or multiple) on-body sensors on multiple segments of human body should be applied for fall recognition. Bourke [26] used two tri-axial accelerometers—one on the trunk and one on the thigh, to find the optimal sensor placement and threshold levels for fall detection. Zhou [27] developed accurate fall-detection solution using two sensors (integrated accelerometer and gyroscope) placed on the chest and thigh, respectively, to recognize standing, bending, sitting, lying down and fall. However, with the development of the methods that use multiple sensors for fall recognition, how to find the optimal locations for sensor placement becomes a matter of high importance. The signals captured at different body segments have different characteristics and thus require different processing algorithms, respectively. This impacts the sensitivity and specificity, and thus also highly impacts the adherence. Existing solutions for pre-impact fall detection used mainly the head (behind the ear) [21], waist [22], trunk/chest (sternum) [23], wrist [24], or hip/thigh [26]. Kangas [28] used accelerometers to find the optimal sensor placements among wrist, waist and head, and showed that the waist and head were the optimal placements for fall detection. Our previous work [13] presented the optimal placement for pre-impact fall recognition using MTx (integrated accelerometer, gyroscope and magnetometer) sensors among five segments of the lower limbs, and showed that the waist was the optimal sensor placement.

## 3. Methods

This study was conducted at Shenzhen Institutes of Advanced Technology (SIAT), Chinese Academy of Sciences, China. The study was approved by the Human Research Ethics Committee of SIAT.

### 3.1. Subjects

It would be very risky to let elderly subjects perform the fall activities in arranged experiments, so eight healthy Asian adult participants aged 28.5 ± 4.3 were recruited from SIAT to perform these activities. The subjects signed an informed consent form prior to taking part in the study.

### 3.2. Sensor Setup

Commercial inertial sensors could be used for recognition and classification of human locomotor activities. This approach has a competitive advantage when applied to non-video monitoring environments. The MTx (The Motion Tracker, Xsens Technologies B.V., The Netherlands) is a small and accurate 3DOF inertial orientation tracker, which could provide drift-free 3D orientation and kinematic data of human body segments: 3D acceleration, 3D rate gyro and 3D Earth-magnetic field. The Xbus Kit (Xsens Technologies B.V., The Netherlands) contains an Xbus Master with Bluetooth wireless link, a wireless receiver and a number of MTx sensor modules. The Xbus Master is a lightweight, portable device that controls multiple MTx modules on the Xbus. Xbus Master and MTx sensor modules are powered by four AA 2,700 mAh batteries which allow continuous operation for at least 3 h.

**Figure 1.**
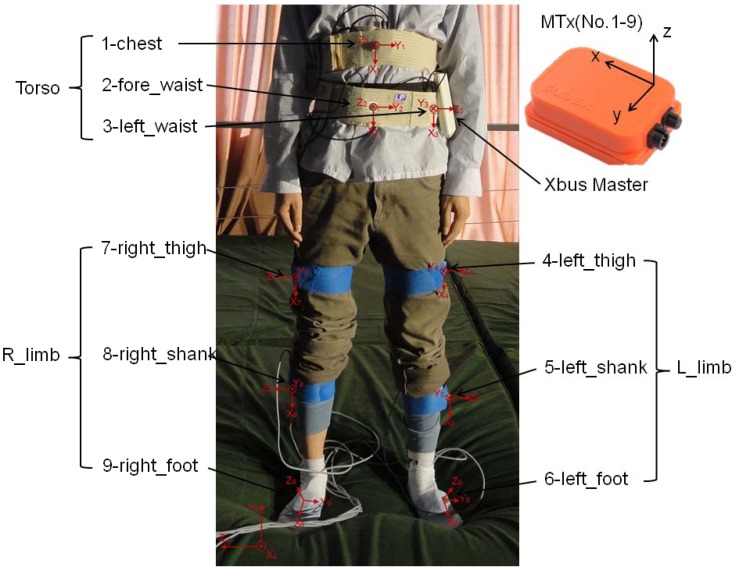
Positions of MTx modules on human body segments.

Nine MTx modules were used to measure the motion of human body segments in the experiments. The arranged positions of MTx modules on the human body were as follows: chest, fore-waist, left waist, left thigh, left shank, left foot, right thigh, right shank, and right foot, and the Xbus Master was attached to the left waist (Figure 1). Five of the MTx modules were connected to the Xbus Master and their data were transmitted to PC wireless by Bluetooth. The other four MTx modules were connected directly to USB port of PC by cable connection. Data sampling frequency was 100 Hz, and the output data had been processed by Kalman filter.

### 3.3. Experimental Procedures

The participants were asked to perform two kinds of activities: Activities of Daily Living (ADL) and Fall Activities (FA). The ADL experimental procedures included calibration, stand-sit-stand, walking, stand-sit-lie; the FA experimental procedures included calibration, forward fall, right-sideway fall and backward fall. Each subject performed each kind of activity three times. In total, 22 trials were arranged in this experiment (Table 1).

### 3.4. Experimentally Measured Data

In the sections above, we assigned a number to each of the trials (*i.e.*, *j* = 1,…,22) and to each of the sensor positions (*i.e.*, *i* = 1,…,9). Based on the arranged numbers, all features of body segment *i* during the trial *j* in the MTx sensor local coordinate frame could be denoted as (x*_ij_*, y*_ij_*, z*_ij_*). So we could describe all kinematic data of the nine MTx sensor modules attached to the human body segments. Once the raw data were filtered by the Xsens Kalman filter, we extracted directly the Acc(x*_ij_*, y*_ij_*, z*_ij_*), Gyr(x*_ij_*, y*_ij_*, z*_ij_*) and Mag(x*_ij_*, y*_ij_*, z*_ij_*) of each MTx for the nine body segments in 22 trials (*i* = 1,…,9; *j* = 1,…,22). Then, the output orientation (roll*_ij_*, pitch*_ij_*, yaw*_ij_*) was calculated by the MTx from the orientation of sensor-fixed coordinate frame (x*_i_*, y*_i_*, z*_i_*, *i* = 1,…,9, see Figure 1) with respect to earth-fixed coordinate frame (x_0_, y_0_, z_0_); the calculated result for the output orientation is of XYZ Earth fixed type. In addition, the resultant values of Acc(*i*, *j*), Gyr(*i*, *j*) and Mag(*i*, *j*) (*i* = 1,…,9; *j* = 1,…,22) could also be calculated by mathematical methods. The calculation equations were defined as follows:

(1) $$\text{Acc}(i,j)=\sqrt{\text{Acc}{\left(x_{ij}\right)}^{2}+\text{Acc}{\left(y_{ij}\right)}^{2}+\text{Acc}{\left(z_{ij}\right)}^{2}}$$

(2) $$\text{Gray}(i,j)=\sqrt{\text{Gray}{\left(x_{ij}\right)}^{2}+\text{Gray}{\left(y_{ij}\right)}^{2}+\text{Gray}{\left(z_{ij}\right)}^{2}}$$

(3) $$\text{Mag}(i,j)=\sqrt{\text{Mag}{\left(x_{ij}\right)}^{2}+\text{Mag}{\left(y_{ij}\right)}^{2}+\text{Mag}{\left(z_{ij}\right)}^{2}}$$

### 3.5. Experimental-Analytical Method

**Figure 2.**
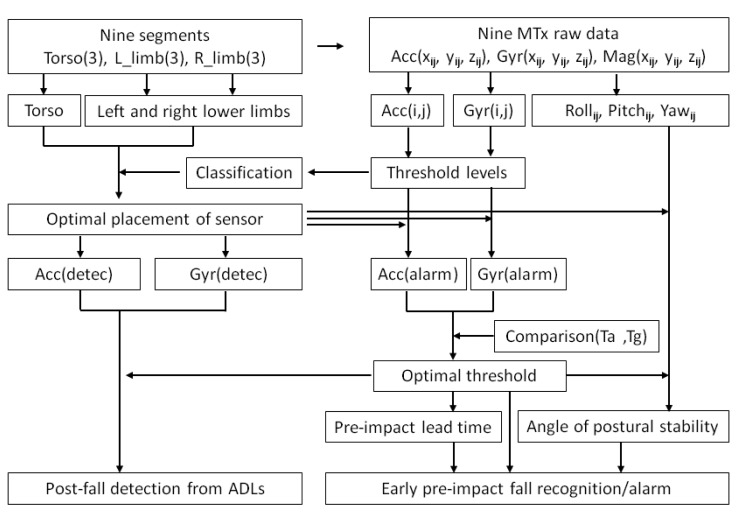
Flow diagram of our method for pre-impact fall recognition/alarm and post-fall detection.

In the section above, the calculation of resultant values of Acc(*i*, *j*), Gyr(*i*, *j*) and orientation (roll*_ij_*, pitch*_ij_*, yaw*_ij_*) was described for each subject for nine sensing segments (*i* = 1,…,9) in a total of 22 trials (*j* = 1,…,22). Based on the threshold levels of the Acc(*i*, *j*) and Gyr(*i*, *j*), we determined the optimal sensor placement by using classification and recognition methods. Then, we determined the thresholds of Acc(alarm), Gyr(alarm) for early pre-impact recognition/alarm as well as Acc(detec), Gyr(detec) for post-fall detection. Subsequently, after comparison of the pre-impact lead time for the acceleration (T_a_) and the pre-impact lead time for the angular rate (T_g_), we determined the optimal threshold level to gain the longest pre-impact lead time. Finally, the critical angle of body postural stability was determined in relation to the pre-impact lead time in different fall activities. The flow diagram of our experimental-analytical method is shown in Figure 2.

## 4. Results and Discussion

The kinematic characteristics of body segments can describe the human locomotion behavior. Firstly, we presented the kinematic features of torso, left and right lower limbs to classify and recognize the ADL and fall activities. The optimal sensor placements for fall recognition/alarm among all investigated segments were determined based on the threshold levels of MTx. Then, by comparison of the pre-impact lead time for the acceleration (T_a_) and the pre-impact lead time for the angular rate (T_g_), we determined the optimal threshold level to gain the longest pre-impact lead time. Finally, the critical angle of postural stability was determined and analyzed relative to the pre-impact lead time for early fall recognition/alarm.

### 4.1. Kinematic Characteristics Used for Fall Recognition

#### 4.1.1. Using the Kinematic Characteristics of Torso for Fall Recognition

**Figure 3.**
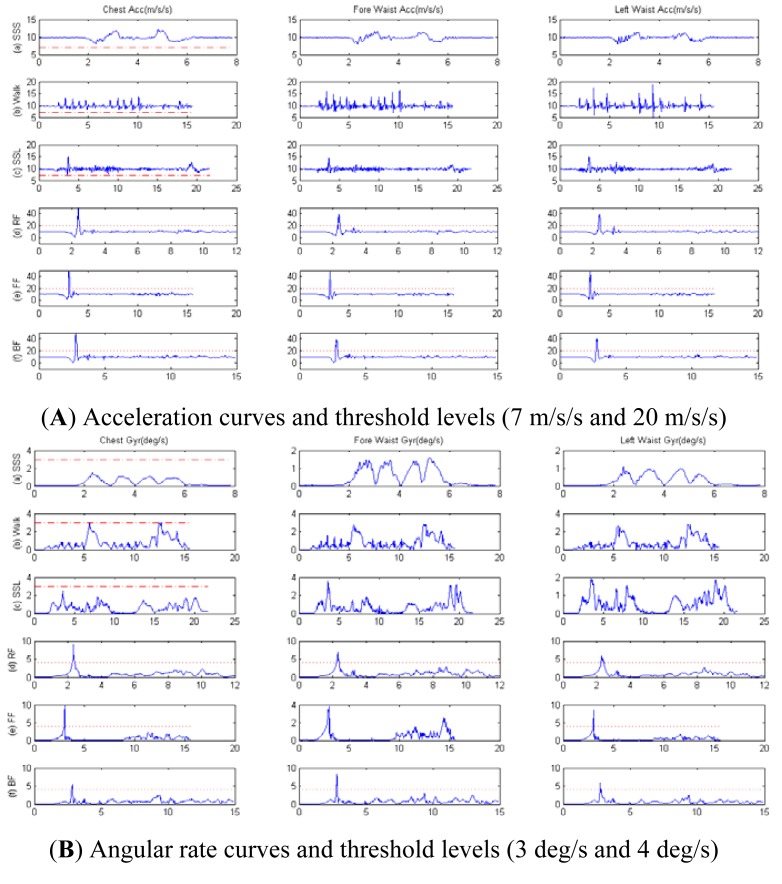
Acceleration (**A**) and angular rate (**B**) curves for torso placements during: (a) SSS, (b) Walking, (c) SSL, (d) RF, (e) FF, (f) BF.

Firstly, the acceleration information for torso was captured during the ADL and fall activities. Figure 3(A) shows that when the resultant acceleration threshold was 20 m/s/s (the red dotted line) the fall activities (RF, FF and BF) for the ADL were recognized with 100% reliability. Based on the results, we discovered that the characteristics of three sensor placements (chest, fore-waist and side-waist) were similar for the three kinds of fall activities. Then, the data for angular rate of torso were obtained as shown in Figure 3(B).

For an angular rate threshold of 4 deg/s (the red dotted line), the reliability obtained for the chest placement was better than the one for the waist for fall recognition/alarm. This showed that only one MTx sensor was enough for successful recognition of a fall event among the ADL, and the chest was the optimal placement area for fall prediction and post-fall detection.

#### 4.1.2. Using the Kinematic Characteristics of Lower Limbs for Fall Recognition

**Figure 4.**
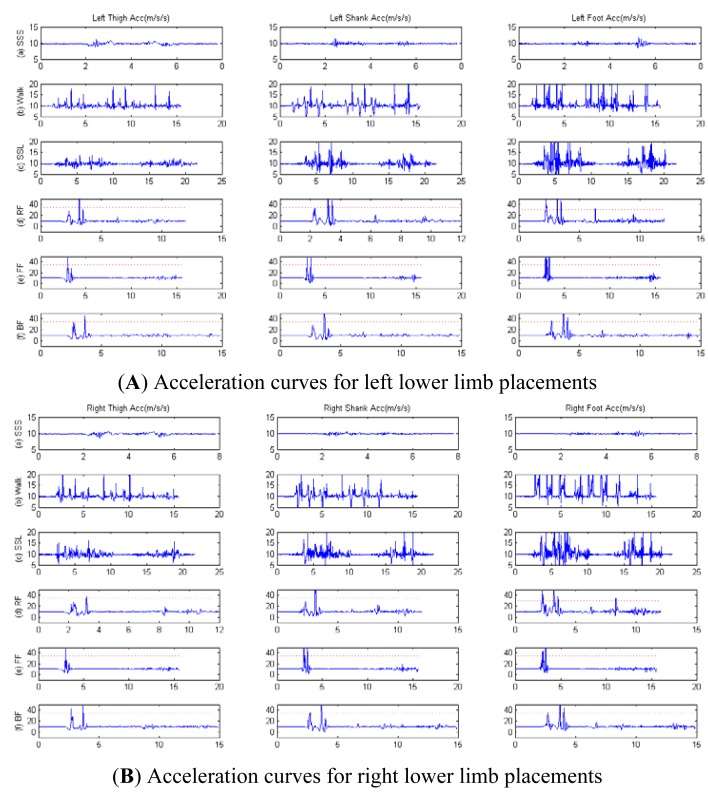

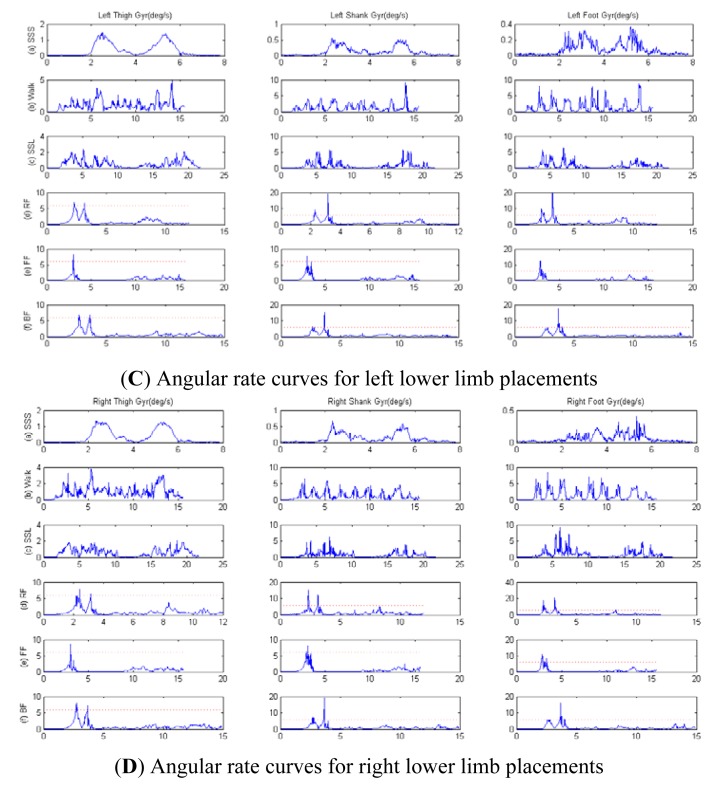
Acceleration (**A,B**) and angular rate (**C,D**) curves for lower limb placements during: (a) SSS, (b) Walking, (c) SSL, (d) RF, (e) FF, (f) BF.

Firstly, the acceleration data from placements on lower limbs were captured during the ADL and fall activities. Figure 4(A,B) show that the optimal threshold to recognize fall events during ADL was 35 m/s/s, and the acceleration curves for the placements on right lower limb and left lower limb were similar. Then, the data received for the angular rate from the placements on the lower limbs were captured as shown in Figure 4(C,D). The angular curves for the placements on the right lower limb and the left lower limb were similar too. With the selected threshold of 6 deg/s for the angular rate (the red dotted line), the placement on the thigh was associated with better fall recognition reliability compared to the placements on shank and foot.

For the same threshold, for walking, SSL and fall activities, the error rates for placements at shank and foot were significant, while we obtained a zero error rate for the thigh placement (a non-zero error rate for walking and SSL activities means that the system falsely reported a fall event while there was no actual fall). Thus, in our experiment, for the lower limbs, to use only one of the thighs (the left or right) as a sensor placement area was the optimal choice for fall prediction and post-fall detection.

#### 4.1.3. Using the Postural Orientation Features for Fall Recognition

**Figure 5.**
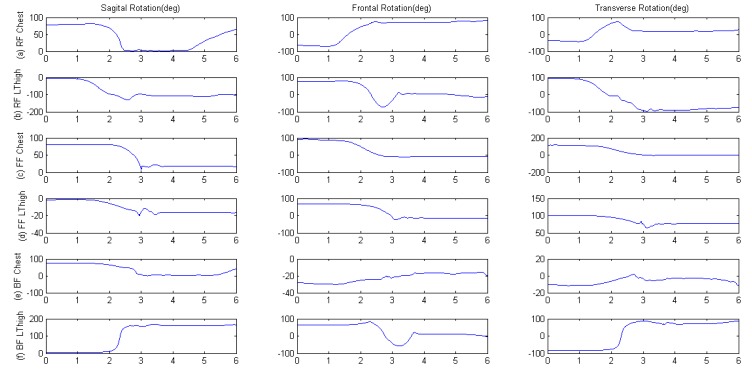
Orientation features of chest and left thigh during: (a,b) RF, (c,d) FF, (e,f) BF.

The postural rotations of chest and left thigh were analyzed during the forward, right-side and backward fall activities. As shown in Figure 5, the impact phase of fall was the time from second 0 to 3, the subsequent phase was the stationary and recovery time for subjects to stand. The results showed that during postural rotation in the different fall directions the chest placement was associated with better reliability compared to the placement on the left thigh segment. The left thigh segment endured hyperextension when the human body impacted the mat bed, so the frontal angle had a larger extension-recovery amplitude as shown in Figure 5(b,f), which could be a reaction based on the self-protection consciousness of subjects. Furthermore, with consideration for the reliability of fall recognition, we chose to use the chest as the optimal placement for the sensor.

### 4.2. Using the Pre-Impact Lead Time and Postural Instability for Pre-Impact Fall Recognition

After the analysis of kinematic features of body segments, we determined that the chest placement was the optimal for fall recognition/alarm. In this section, we determined the optimal pre-impact lead time and the critical angle of body postural stability, and further analyzed the preventive and protective mechanisms and their relation with the trade-off between the lead time and the angle of body postural stability in different falls.

#### 4.2.1. Determination of the Optimal Pre-Impact Lead Time Using the Acceleration and Angular Rate

In this section we analyzed the thresholds of acceleration and angular rate of chest segment in order to gain the longest pre-impact lead time.

In Figure 3(A)(a,b,c) we can see that all values of acceleration were higher than the threshold of 7 m/s/s (the red dash-dotted line) during the SSS, Walking and SSL activities. Thus, we chose the acceleration value of 7 m/s/s as a threshold for early pre-impact fall recognition/alarm during ADL and fall activities.

In Figure 3(B)(a,b,c) we can see that all values of angular rate were lower than the threshold of 3 deg/s (the red dash-dotted line) during the SSS, Walking and SSL activities. So the angular rate value of 3 deg/s could serve as a threshold for early pre-impact fall recognition/alarm during ADL and fall activities.

**Figure 6.**
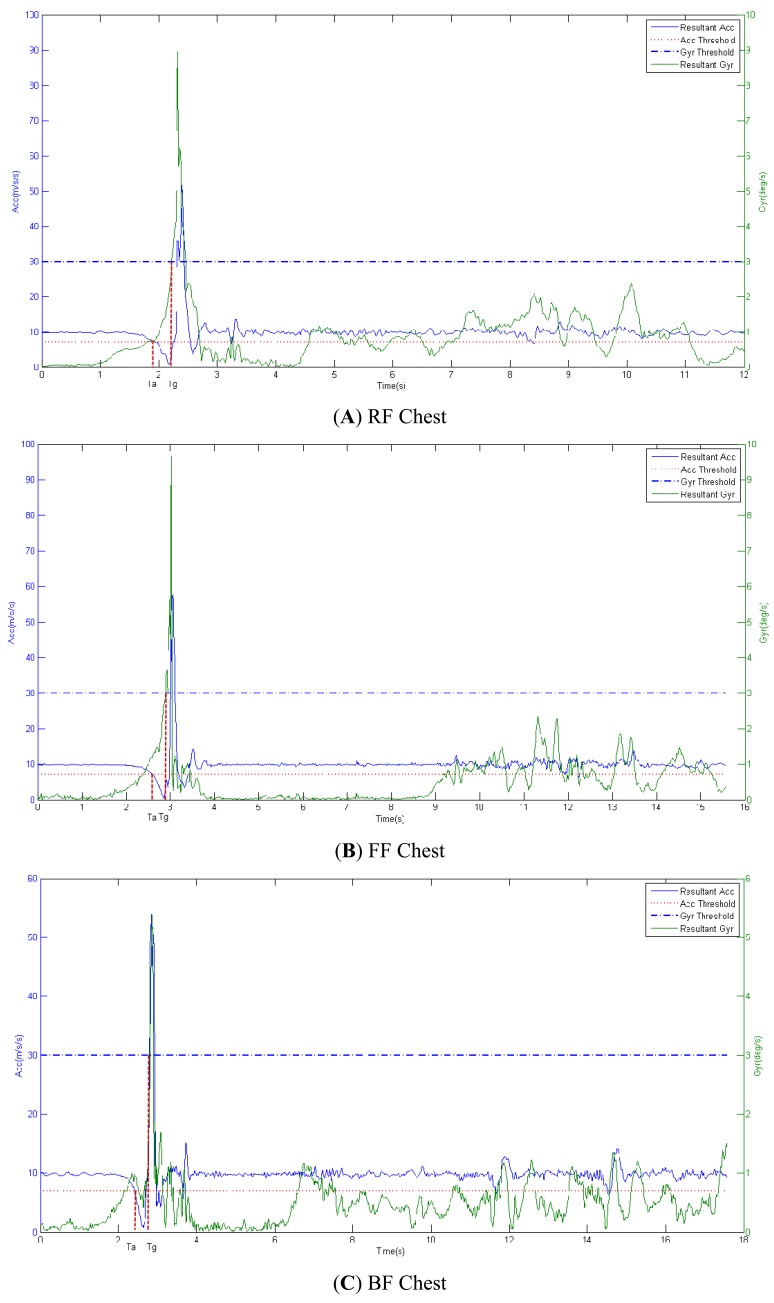
Determination of optimal pre-impact lead time using the threshold levels for acceleration and angular rate of chest segment. (**A**) RF; (**B**) FF; (**C**) BF.

We estimated the optimal pre-impact lead time using the above chosen threshold levels of 7 m/s/s Acc(alarm), and 3deg/s Gyr(alarm). In Figure 6 is shown that the pre-impact lead time for acceleration (T_a_) was always earlier than the pre-impact lead time for angular rate (T_g_) regardless of the direction of the fall (RF, FF or BF). Thus, the pre-impact lead time based on the threshold for acceleration was determined as the optimal one and it was the longest in our experiment for early pre-impact fall recognition/alarm.

#### 4.2.2. Methods for Calculation of Pre-Impact Lead Time and Angle of Body Postural Stability

Based on the threshold levels for acceleration, the pre-impact lead time and angle of postural stability for chest placement were determined during the three different fall activities. The acceleration threshold of 7 m/s/s was chosen as a base point to determine the pre-impact alarm time (T_1_). The impact time of fall was denoted as T_2_. The pre-impact lead time (T) was defined as follows:

(4) $$T=T_{2}-T_{1}$$

The initial angle of postural stability for chest placement in fall-side plane was defined as *θ*_1_. The largest angle, related to angular motion of the body in order to avoid falling down we called critical angle of postural stability and denoted it as *θ*_2_; this angle is based on the pre-impact alarm time (T_1_). The angle of postural stability (*θ*) was defined as follows:

(5) $$\theta =\left|\theta_{2}-\theta_{1}\right|$$

**Figure 7.**
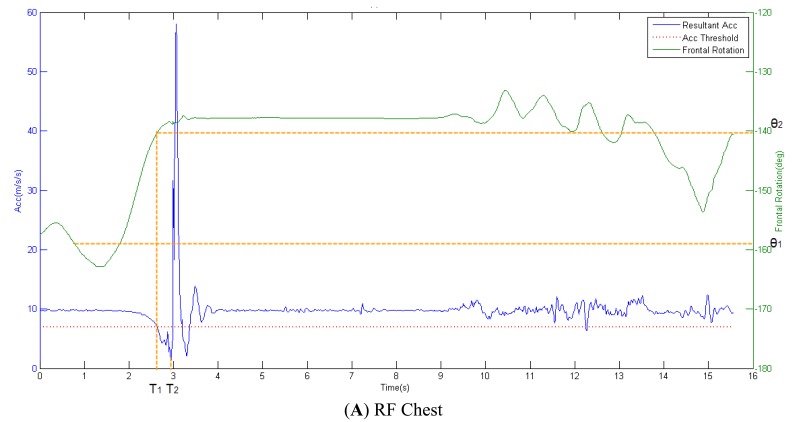

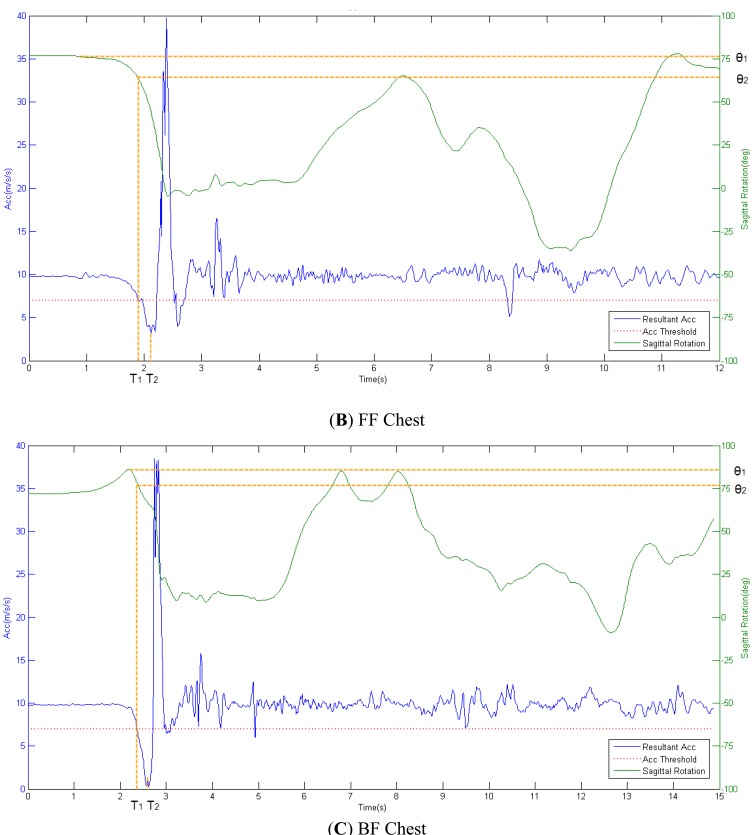
Clarification of the calculation method used to find the relation between pre-impact lead time and angle of postural stability for chest placement. (**A**) RF; (**B**) FF; (**C**) BF.

Figure 7 shows the resultant acceleration related to the rotation angle of fall-side plane during three fall activities.

#### 4.2.3. Analysis of Pre-impact Lead Time Relative to Fall Postural Instability

**Figure 8.**
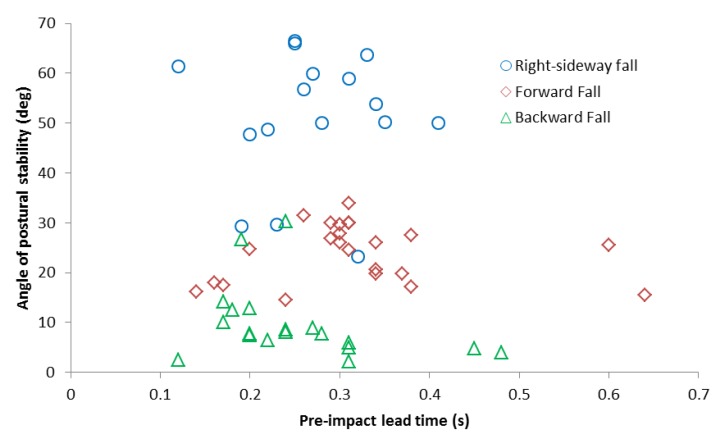
Pre-impact lead time *vs.* the angle of postural stability for chest placement for all valid trials of subjects.

Figure 8 is based on the calculations above. It shows the relation between angle of postural stability and pre-impact lead time for the chest placement, for all of the three fall activities and all valid trials of subjects. In our experiment, the longest pre-impact lead time obtained was 640 ms in forward fall. In Table 2 are given the average pre-impact lead time (T) and average angle of postural stability (*θ*).

Experimental results given in Table 2 showed that the right-sideway fall led to the largest angle of postural stability; the forward fall led to the longest pre-impact lead time; the backward fall led to the shortest lead time and smallest angle of postural stability. This conclusion implied that among the three fall activities the backward fall was the toughest to avoid due to the inability of the self-protective mechanism to compensate it. That is why we suggested that people should be careful to avoid the backward fall as much as possible. Results also implied that, in contrast with the backward fall, the forward and sideway falls could be easily compensated by postural adjustment of body segments.

## 5. Conclusions

This study presented the study and implementation of a pre-impact fall recognition/alarm method for free-direction fall activities, based on an understanding of the pre-impact lead time of fall and angle of postural stability, using an inertial body sensor network. The inertial sensor modules were attached to nine human body segments in order to determine the optimal segments for sensor placements for fall recognition/alarm and explore the average pre-impact lead time and the average critical angle of postural stability for pre-impact fall alarm in three kinds of fall activities.

After analyzing the kinematics of torso, left and right lower limbs, we found that only one sensor was enough on the torso, and the chest placement was the optimal choice; meanwhile, for the lower limbs, the thigh sensor showed best performance for fall recognition/alarm. Then reliability analysis of fall recognition was performed and the results showed that the chest placement was associated with better fall recognition reliability compared to the thigh placement, so we suggested that the chest segment should be regarded as the optimal and reasonable placement.

In order to gain the longest pre-impact lead time, we used the acceleration threshold value of 7 m/s/s and the angular rate threshold value of 3 deg/s. The results showed that the pre-impact lead time for the acceleration (T_a_) was always earlier than the pre-impact lead time for the angular rate (T_g_), regardless the direction of the fall, so the acceleration threshold levels were determined for fall recognition/alarm using the chest sensor placement. The lower threshold (7 m/s/s), Acc(alarm) was used for pre-impact recognition/alarm, while the higher threshold (20 m/s/s), Acc(detec) was used to detect post-fall events. We used the relation between the average pre-impact lead time and the average critical angle of postural stability to optimize our system for early fall recognition/alarm. The longest average lead time was 329 ± 21 ms during forward falls, and the largest average angle of postural stability was 49.9 ± 4.1 degrees during sideway falls. The result implied that, due to the specific trade-off between the pre-impact lead time and the angle of postural stability for each kind of activity, the forward and sideway falls could be easily prevented, while this was not the case for backward falls.

It should be clear that all experiments were performed by healthy young subjects under mat-bed cushioned impact conditions. Thus the experimental results could not completely represent the real conditions of elderly falls. In the future, in order to acquire data of real elderly falls in a natural environment, we will mount a chest placement sensor on people who are at high risk of falls, and thus conduct continuous 24-h monitoring of fall events.

## Figures and Tables

**Table 1.** Experimental procedures used for ADL and FA for each subject in this study.

**Activities**		**Procedures of Trials**
Activities of Daily Living (ADL)	(a) Stand-Sit-Stand (SSS)	Trial 1 (standing postural calibration). All nine MTx sensor modules were attached. Calibration for each subject was needed to be performed in standing posture prior starting a new group of activities. Trials 2–4 (SSS_1, SSS_2, SSS_3). Each subject performed the stand-sit-stand activity. The chair height was 50 cm.
	(b) Walking (Walk)	Trial 5 (walking calibration). The same as Trial 1. Trials 6–8 (W_1, W_2, W_3). Each subject walked straight forward for 5 m, turned around and walked back to the starting point.
	(c) Stand-Sit- Lie (SSL)	Trial 9 (SSL calibration). The same as Trial 1 Trials 10–12 (SSL_1, SSL_2, SSL_3). Each subject performed the following activity: from standing position to sit on the edge of the mat (mat height was50 cm); then starting from the same sit position to lie down to the mat surface.

Fall Activities (FA)	(a) Right-sideway Fall (RF)	Trial 13 (fall calibration). The same as Trial 1. Trials 14–16 (RF_1, RF_2, RF_3). Each subject performed the following activity: starting from standing position, the subject performed a fall to subject's right-side down to the mat surface.
	(b) Forward Fall (FF)	Trials 17–19 (FF_1, FF_2, FF_3). Each subject performed the following activity: starting from standing position to fall down forward to the mat surface.
	(c) Backward Fall (BF)	Trials 20–22 (BF_1, BF_2, BF_3) Each subject performed the following activity: starting from standing position, the subject performed a backward fall down to the mat surface.

**Table 2.** Average pre-impact lead time and angle of postural stability for chest placement, all subjects and all fall activities.

**Fall Activities**	**Pre-Impact Lead Time (ms)**	**Angle of Postural Stability (deg)**
Right-sideway Fall (RF)	265 ± 35	49.9 ± 4.1
Forward Fall (FF)	329 ± 21	23.9 ± 3.3
Backward Fall (BF)	257 ± 36	9.9 ± 2.5
